# Supplementary material for: Assessment of Cold Atmospheric Pressure Plasma (CAPP) Treatment for Degradation of Antibiotic Residues in Water
Source: Antibiotics (Basel). 2023 Jun 28;12(7):1115. doi: 10.3390/antibiotics12071115 (PMC10376056; doi:10.3390/antibiotics12071115)
Supplement: Supplementary file 1 [file antibiotics-12-01115-s001.zip › antibiotics-2465012-supplementary.pdf]

Supplementary Materials:

Table S1. Results of the WRL validation studies in milk where: accuracy is expressed as percentage of spiked concentration versus measured concertation; reproducibility is expressed as a covariance of concentration measurement accuracy; recovery is a ratio between the amount of analyte spiked before and after the extraction at calibration level two (CL2) and four (CL4); matrix effect is the ratio between the analye's measured response in blank matrix (n = 20) spiked after the extraction and equivalent concentration in reconstitution solvent with relative standard deviation (RSD), negative values indicating ios suppression.

Analyte	MRL [ng/g]	Linear range [ng/g]	R^2	0.5MRL (n=6)		MRL (n=6)		1.5MRL (n=6)		CCα [ng/g]	CCβ [ng/g]	Recovery [%]		Matrix effect	RSD (n=20)
				Accuracy	Repeatability	Accuracy	Repeatability	Accuracy	Repeatability			CL2	CL4		
Amoxicillin	4	1-10	0.998	99%	10%	99%	10%	99%	8%	4.47	5.04	52	53	9%	7%
Amplicillin	4	1-10	0.997	96%	10%	96%	8%	96%	7%	4.56	5.27	39	41	-39%	8%
Penicillin G	4	1-10	0.999	105%	9%	103%	9%	105%	8%	4.57	5.26	93	95	-97%	11%
Nafcilin	30	7.5-75	0.999	99%	4%	99%	4%	100%	3%	32.3	34.1	95	93	-16%	5%
Oxacillin	30	7.5-75	0.999	97%	8%	92%	7%	92%	6%	33.4	37.3	101	98	-16%	9%
Cloxacillin	30	7.5-75	0.999	97%	4%	96%	6%	99%	4%	32.1	34.7	96	95	-9%	6%
Dicloxacillin	30	7.5-75	0.999	93%	5%	92%	7%	91%	6%	33.4	37.0	98	97	-10%	7%
Cephalexin	100	25-250	0.999	105%	5%	103%	5%	102%	4%	109	118	50	47	-18%	7%
Cefquinome	20	5-50	0.999	91%	9%	90%	15%	88%	11%	24.5	30.5	79	74	-43%	10%
Cefoperazone	50	12.2-125	0.999	87%	8%	86%	10%	96%	9%	53.3	62.3	92	87	-37%	7%
Cefacetrile	125	31.2-312	0.999	98%	6%	99%	6%	96%	6%	140	154	75	71	-3%	7%
DAC	60	15-150	0.998	111%	8%	112%	10%	115%	14%	71.5	93.0	63	58	-45%	6%
DCCD	50	12.2-125	0.998	96%	4%	95%	9%	95%	10%	57.2	67.9	33	31	-30%	7%
